# Erratum for Balthazar et al., “A laboratory-based predictive pathway for the development of *Neisseria gonorrhoea*e high-level resistance to corallopyronin A, an inhibitor of bacterial RNA polymerase”

**DOI:** 10.1128/spectrum.01406-24

**Published:** 2024-07-01

**Authors:** Jacqueline T. Balthazar, Daniel Golparian, Magnus Unemo, Timothy D. Read, Miriam Grosse, Marc Stadler, Kenneth Pfarr, Andrea Schiefer, Achim Hoerauf, Jennifer L. Edwards, Dmitry G. Vassylyev, William M. Shafer

## ERRATUM

Volume 12, no. 6, e00560-24, 2024, https://doi.org/10.1128/spectrum.00560-24. Page 3, paragraph 1: Reference 13 should be reference 6.

Page 9, paragraph 6: Reference 13 should be reference 14, and reference 14 should be reference 6.

